# Long-Term Impact of Battle Injuries; Five-Year Follow-Up of Injured Dutch Servicemen in Afghanistan 2006-2010

**DOI:** 10.1371/journal.pone.0115119

**Published:** 2015-02-02

**Authors:** Rigo Hoencamp, Floris J. Idenburg, Thijs T. C. F. van Dongen, Loes G. M. de Kruijff, Eelco P. Huizinga, Marie-Christine J. Plat, Erik Hoencamp, Luke P. H. Leenen, Jaap F. Hamming, Eric Vermetten

**Affiliations:** 1 Department of Surgery, Leiden University Medical Centre, Leiden, The Netherlands; 2 Department of Surgery, Medical Centre Haaglanden, The Hague, The Netherlands; 3 Department of Surgery, University Medical Centre, Utrecht, The Netherlands; 4 Department of Physiatrist, Rehabilitation Center de Hoogstraat, Utrecht, The Netherlands; 5 Force Health Protection, Expert Centre Force Health Protection Ministry of Defense, Utrecht, The Netherlands; 6 Leiden University Medical Centre, Leiden, The Netherlands; 7 Leiden University Medical Centre, Military Mental Health Research, Utrecht, The Netherlands; Univ of Toledo, UNITED STATES

## Abstract

**Objectives:**

Units deployed to armed conflicts are at high risk for exposure to combat events. Many battle casualties (BCs) have been reported in the recent deployment to Afghanistan. The long-term impact of these combat injuries, at their five-year end point, is currently unknown. To date, no systematic inventory has been performed of an identified group of BCs in comparison to non-injured service members from the same operational theatre.

**Design:**

Observational cross-sectional cohort study.

**Setting:**

Open online survey among Dutch BCs that deployed to Afghanistan (2006–2010).

**Participants:**

The Dutch BCs (n = 62) were compared to two control groups of non-injured combat groups (battle exposed [n = 53], and non-battle exposed [n = 73]).

**Main Outcome Measures:**

Participants rated their impact of trauma exposure (Impact of Events [IES]), post deployment reintegration (Post Deployment Reintegration Scale [PDRS]), general symptoms of distress (Symptom Checklist 90 [SCL-90]), as well as their current perceived quality of life (EuroQol-6D [EQ-6D]). Also cost effectiveness (Short From health survey [SF-36]) and care consumption were assessed (Trimbos/iMTA questionnaire).

**Results:**

Over 90% of BCs were still in active duty. The mean scores of all questionnaires (IES, EQ-6D, SF-36, and SCL-90) of the BC group were significantly higher than in the control groups (p<0.05). The PDRS showed a significantly lower (p<0.05) outcome in the negative subscales. The mean consumption of care was triple that of both control groups. A lower score on quality of life was related to higher levels of distress and impact of trauma exposure.

**Conclusions:**

This study showed a clear long-term impact on a wide range of scales that contributes to a reduced quality of life in a group of BCs. Low perceived cost effectiveness matched with high consumption of care in the BC group in comparison to the control groups. These results warrant continuous monitoring of BCs.

## Background

Military medicine covers a large area of interest, including battle (combat) related acute medical and surgical interventions, but also the long-term physical and psychological wellbeing of service members. Research in military medicine is essential to enable justification for new doctrines, practices and management guidelines. This field is fueled with balancing design imperatives, calls for operational sustainability, military ethics, and optimizing quality of life (QOL) for the service members in the aftermath of service. Recently, we described the short term outcome and the impact of events on the direct circle around battle casualties (BCs) of service members serving in Task Force Uruzgan (TFU), that deployed as part of the North Atlantic Treaty Organization (NATO) led International Security Assistance Force (ISAF) mission in Southern Afghanistan [1–2]. Many studies report on the immediate impact on general health [3–8], and long-term studies tend to focus solely on mental health impact [9–10] of deployments. To date, no systematic assessment has been performed evaluating the long-term follow-up of an identified group of injured service members in comparison to an equal group of non-injured service members from the same operational theatre. Awareness of and insight into the short and long-term impact of combat injuries can provide opportunities for case orientated health surveillance programs. The aim of this study was to compare the five-year follow-up of QOL and health care consumption of injured service members to a comparable group of non-injured service members. Possible associations between type and severity of injury and long-term outcome of the injured service members were explored. We also tried to identify possible points of improvement in post-deployment treatment and re-integration, by identifying the predictive value of combat related factors (injury, deployment effects, danger to life) for QOL.

## Material and Methods

### Study design and participants

This observational cohort study was conducted among Dutch service members during the period 2006–2010. In these years, 12 brigades (~17,000 service members) [3] were deployed to multinational base Tarin Kowt (MBTK) in Southern Afghanistan, in 4–5 month periods, as part of TFU. The participants consisted of three groups: (1) service members that were injured in theatre, labeled as BCs, (2) non-injured active combatants from the same combat units (control group 1 [CG1]), and (3) non-injured service members, with a staff function on MBTK (control group 2 [CG2]). Battle casualties were defined as service members being injured as a direct result of hostile action, sustained in combat or sustained going to or coming from a combat mission. The BCs were selected from a general digital admission database of the Ministry of Defense (MOD), where they fitted the criteria ‘BC between August 2006 and August 2010’. The following variables were used as injury specific information: mechanism of injury (MOI), anatomical distribution of wounds (AD), and Injury Severity Score (ISS). The control groups were randomly selected by an independent employee from the department of epidemiology of the MOD. The only exclusion criterion in the control groups was sustaining a battle injury. All identified service members were requested to complete an online questionnaire in the last quarter of 2013 (mean ~ five years after deployment). If necessary they received two digital reminders and two reminders by telephone. The participants were divided into five rank groups namely; junior enlisted (E1-E4), senior enlisted (E5-E9), warrant officers (WO1-WO2), junior officers (O1-O3), and senior officers (O4-O10). As socio-demographic characteristics were measured: sex, age, marital status, and educational level.

### Assessment

The survey contained five domains: (1) the Impact of Event Scale (IES) [11], (2) the Post Deployment Reintegration Scale (PDRS) [12–13], (3) the Symptom Checklist 90 (SCL-90) [14–16], (4) Quality of Life using the EuroQol-6D (EQ-6D) [17], the 36-item Short Form health survey (SF-36) [18], and (5) the modified Trimbos/iMTA questionnaire for Costs associated with Psychiatric Illness (TIC-P) [19]. All assessments were self-reported.

The IES [11] consists of a 22-item measurement that assesses traumatic stress. Responses are given on a 5-point scale, scoring 0 (not at all) to 4 (extreme), and render a total score (zero to 88), subdivided in the following subscales: intrusion (INT), avoidance (AVO), and hyper arousal (HAR) [11].

The PDRS [12] contains 36 items, and is a multidimensional measure of post deployment reintegration experiences/attitudes that is designed to reflect a continuum experience of military personnel in several domains (Work negative [WN]; Work positive [WP]; Family negative [FN]; Family positive [FP]; Personal negative [PN], and Personal positive [PP]). Each domain is split into a positive and negative subscale (score 0–5). On negative subscales higher scores indicate more negative attitudes, and on positive subscales higher scores indicate more positive attitudes [13].

The SCL-90 [14–16], containing 90 questions with a 5-point rating scale (ranging from 1 [not at all] to 5 [extreme]), are used to assess physical and psychological symptoms of distress. Outcome scores are divided into nine symptom subscales: anxiety (ANX, range 10–50), agoraphobia (AGO, range 7–35), depression (DEP, range 16–80), somatization (SOM, range 12–60), insufficient thinking and handling (IN, range 9–45), distrust and interpersonal sensitivity (SEN, range 18–90), hostility (HOS, range 6–30), sleeping disorders (SLE, range 3–15), and a rest subscale (REST, range 9–45). The total score (SCL-90-TOT, range 90–450) is calculated by adding the scores of the subscales.

The EQ-6D^17^ questionnaire is a concise utility index, designed to measure health-related quality of life and health preferences, using a visual analogue scale. The SF-36 [18] is a survey of patient health, and is a measure of health status, commonly used in health economics as a variable in the quality-adjusted life year calculation to determine the cost-effectiveness of a health treatment. The SF-36 consists of eight scaled scores (vitality, physical functioning, bodily pain, general health, physical role functioning, emotional role functioning, social role functioning, and mental health), which are the weighted sums of the questions in their section. Each scale is directly transformed into a 0–100 scale (i.e. zero is maximum disability and 100 is no disability).

For calculating the total direct medical costs, the Trimbos/iMTA questionnaire for Costs associated with Psychiatric Illness (TiC-P) [19] was used. The scale allows to assess general utilization of medical treatment such as the number of contacts with the general practitioner and multiple other care providers (e.g. medical specialist, physical therapist, and psychologist) during the last six months, including stay or treatment in university, psychiatric, or general hospitals. The costs were estimated using the Dutch guidelines for cost calculations in health care [20]. Reference unit prices from 2006 of the corresponding health care services were applied [21].

### Statistical analysis

In addition to demographics, we used post deployment questionnaires and, only for the BCs, information about the injury (Table 1). Continuously distributed variables were summarized by the mean value and standard deviation (SD). Absolute and relative frequencies were used to describe nominal and ordinal variables.

The Kruskall Wallis test was used to identify differences in the questionnaire outcomes between the BC group and both control groups. Relations between quality of life (EQ-6D) and psychological- and physical distress, traumatic stress, and post deployment reintegration, were determined with Pearson’s r correlation test. Relations in the BC group between QOL, age and ISS score were also calculated with a Pearson correlation. Due to measurement level, the relations between EQ-6D versus AD (lower & upper extremity, truncal, head & neck, and combined injuries) and rank were determined with univariate regression analyses. Statistical analyses were performed using a computerized software package, SPSS (Version 20, IBM Corporation, Armonk, New York). This study was approved by the MOD and the Institutional Review Board and the Medical Ethics Committee of Leiden University, the Netherlands.

**Table 1:** Variables analyzed.

Variables		
All groups		
Demographics		
Age	Sex	Rank
Marital status	Educational status*	Number of deployments
Post deployment		
IES	PDRS	SCL-90
EQ-6D	SF-36	Modified TIC-P
Battle casualties		
Injury date	Mechanism of injury	AD
ISS		

MOI indicates Mechanism of injury

AD: Anatomical Distribution

ISS: Injury Severity Score

*Educational status was divided into three groups: low (no formal education, elementary school, lower vocational education or lower general secondary education), middle (middle general secondary education), and high (college or university).

### Ethics approval

This study was approved by the Ministry of Defense (MOD), the Institutional Review Board and the Medical Ethics Committee of Leiden University, the Netherlands (p11.184).

## Results

**Fig 1:**
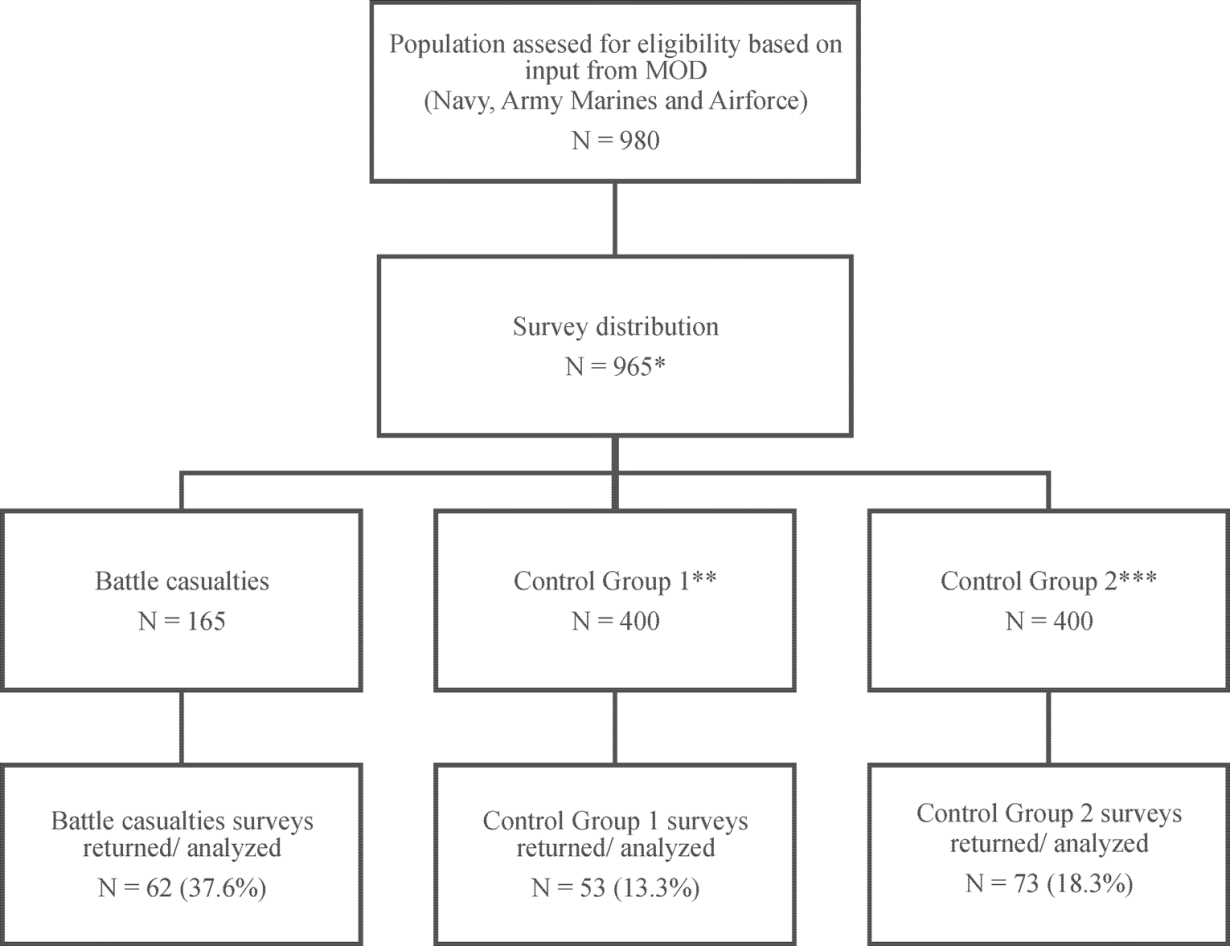
CONSORT diagram for questionnaire Dutch battle casualties and control group deployed to Southern Afghanistan.

All questionnaires were distributed online. Of the 965 questionnaires that were distributed (Fig. 1), 165 were distributed to BCs, 400 to CG1, and 400 to CG2. Respectively, the response rate in the BC group was 38% (62/165, [of this group 53% were repatriates, 48/90], [19% returned to duty [RTD], 14/75]), 13% (53/400) in CG1, and 18% (73/400) in CG2. Almost eighty percent (149/187) of the participants was aged between 20 and 40. Ninety percent of the BCs were still in active duty, 92% of CG1, and 88% of CG2. The mean number of deployments of the participants was 3 (range 1–8). Demographics of the BC group closely matched composition of CG1. Respondents in CG2 were significantly older and higher educated than in the first two groups (see Table 2).

**Table 2:** Demographics of battle casualties and both control group.

Characteristic during deployment	BC	CG1	CG2
	N = 62	N = 53	N = 73
**Age**, mean (range)	25.6 (18–49)	28.1 (19–49)	37.2 (19–58)
**Sex** (%)			
Male	61 (98.4)	50 (94.3)	65 (89.0)
Female	1 (1.6)	3 (5.7)	8 (11.0)
**Marital Status** (%)			
Married/ Registered partner	20 (32.3)	27 (50.9)	43 (58.9)
Relationship	28 (45.2)	17 (32.1)	9 (12.3)
Single	14 (22.6)	9 (17.0)	21 (28.8)
Divorced	0	1 (0.2)	3 (0.4)
Widow	0	0	1 (0.1)
**Active duty** (%)	56 (90.3)	49 (92.4)	64 (87.7)
**Rank** (%)			
E1-E4	43 (69.4)	23 (4.3)	10 (13.7)
E5-E9	12 (19.0)	16 (3.0)	23 (31.5)
WO1-WO3	1 (1.6)	1 (0.2)	7 (9.6)
O1-O3	6 (9.5)	12 (2.3)	22 (30.1)
O4-O10	0	1 (0.2)	11 (15.1)
**Number of deployments**, mean (range)	2.5 (1–7)	2.8 (1–7)	2.9 (1–8)
**Educational status** (%)			
Low	1 (1.6)	1 (0.2)	4 (5.5)
Middle	58 (92.1)	43 (81.1)	44 (60.3)
High	3 (4.8)	9 (17.0)	25 (34.2)

BC indicates battle casualty

CG1: control group 1

CG2: control group 2

N: number

E1-E4: junior enlisted

E5-E9: senior enlisted

WO1–WO3: warrant officers

O1–O3: junior officers

O4–O10: senior officers.

The MOI was in 96.7% (60/62) explosions (80.6% [50/62] IEDs) and in 2.3% (2/62) small arms fire. The AD was as follows: 30.6% (19/62) lower extremity, 4.8% (3/62) upper extremity, 9.7% (6/62) truncal, 9.7% (6/62) head & neck, and 45.1% (28/62) combined injuries. The mean ISS was 10.4 (SD 9.8). In the sub-analysis within the BC group, using the Pearson’s test, there were no significant correlations between the QOL and the continuous variables ISS (r -.19; p = 0.20) and age (r -.27; p = 0.06),. Univariate regression turned out that rank (p = 0.004) was positively associated with QOL, where MOI (p = 0.20) and AD (p = 0.10) were not. The care consumption in the subgroup of lower extremity injuries (single and combined) was significantly higher (p = 0.03) compared to the other combat injuries. There was no significant relation (p = 0.18) with QOL and care consumption.

The mean IES scores were respectively in the BC group 15.9 (SD 18.8), CG1 5.1 (SD 9.6), and CG2 3.7 (SD 7.1). Significant differences (p<0.05) between the three groups were found on the IES as well as the PDRS. This was also true for assessment of the subscales (intrusion, avoidance, and hyper arousal) (p<0.05). The PDRS also showed a significant different outcome (p<0.05) in all negative subscales, when comparing the 3 subgroups (Table 3).

**Table 3:** Scores of IES, PDRS, SCL-90, EQ-6D and TIC-P per subgroup.

Variable Mean	BC	CG1	CG2	P value
(SD)	N = 62	N = 53	N = 73	
IES*	15.9 (18.8)	5.1 (9.6)	3.7 (7.1)	<0.0001ɸ
INT	6.4 (7.6)	2.5 (4.1)	1.6 (3.2)	<0.0001ɸ
AVO	4.0 (5.9)	1.5 (3.7)	0.9 (2.0)	<0.0001ɸ
HAR	5.5 (6.6)	1.1 (2.5)	1.2 (2.3)	<0.0001ɸ
PDRS**				
WP	3.7 (0.6)	3.5 (0.7)	3.5 (0.8)	0.36
WN	2.8 (1.1)	2.4 (1.1)	2.1 (0.8)	0.001ɸ
FP	3.1 (0.9)	3.0 (0.8)	3.0 (0.9)	0.95
FN	2.3 (0.9)	2.0 (0.8)	1.8 (0.8)	0.003ɸ
PP	3.3 (0.8)	3.0 (1.0)	3.2 (0.9)	0.43
PN	2.4 (1.0)	1.9 (0.9)	1.8 (0.7)	0.001ɸ
SCL-90***	135.5 (46.7)	107.4 (22.2)	107.3 (25.6)	<0.0001ɸ
ANX	14.5 (5.8)	11.2 (2.0)	11.6 (3.9)	<0.0001ɸ
AGO	9.0 (3.3)	7.5 (1.1)	7.6 (1.4)	<0.023ɸ
DEP	23.4 (8.4)	19.2 (4.7)	19.5 (6.9)	<0.005ɸ
SOM	19.1 (7.6)	14.9 (4.8)	14.6 (3.3)	<0.0001ɸ
IN	16.6 (7.1)	11.7 (3.8)	11.2 (3.2)	<0.0001ɸ
SEN	25.2 (8.4)	21.6 (5.4)	21.7 (6.0)	0.005ɸ
HOS	9.6 (4.6)	7.2 (1.7)	7.0 (1.5)	0.001ɸ
SLE	5.6 (3.0)	3.8 (1.3)	4.2 (1.9)	0.002ɸ
REST	12.4 (4.2)	10.3 (2.4)	10.0 (1.9)	<0.0001ɸ
EQ-6D	77.6 (17.2)	86.7 (12.8)	86.0 (11.7)	0.002ɸ
SF-36****				
PF	78.6 (22.6)	97.4 (7.9)	93.6 (15.2)	<0.0001ɸ
SF	79.7 (21.0)	93.4 (15.2)	91.2 (16.5)	<0.0001ɸ
RP	69.6 (41.9)	93.4 (19.0)	92.8 (20.1)	<0.0001ɸ
RE	73.2 (40.0)	86.8 (30.9)	92..8 (19.7)	0.004ɸ
MH	72.4 (18.3)	83.0 (13.7)	82.1.7 (14.4)	0.003ɸ
VT	61.9 (19.7)	75.7 (16.8)	72.7 (17.1)	0.001ɸ
BP	74.1 (24.7)	90.8 (13.5)	89.3 (14.9)	<0.0001ɸ
GP	67.4 (19.0)	77.0 (17.0)	74.8 (17.6)	0.02ɸ
TIC-P*****	486.8 (1153.5)	162.9 (197.3)	166.1 (328.8)	0.02ɸ

BC indicates battle casualty

CG1: control group 1

CG2: control group 2

SD: standard deviation

N = numberɸ significant difference (p<0.05) using the Kruskall Wallis test.

*Subscales IES

INT: intrusion; AVO: avoidance

HAR: hyper arousal.

**Subscales PDRS

WP: Work positive

WN: Work negative

FP: Family positive

FN: Family negative

PP: Personal positive

PN: Personal negative.

***Subscales SCL-90

ANX: anxiety

AGO: agoraphobia

DEP: depression

SOM: somatization

IN: insufficient thinking and handling

SEN: distrust and interpersonal sensitivity

HOS: hostility

SLE: sleeping disorders

REST: rest subscale.

****Subscales SF-36

PF: Physical functioning

SF: Social functioning

RP: Role physical

RE: Role emotional

MH: Mental health

VT: Vitality

BP: Bodily pain

GH: General Health.

***** Costs in euros.

The mean overall SCL-90 scores were respectively in the BC group 135.5 (SD 46.7), CG1 107.4 (SD 22.2) and CG2 107.3 (SD 25.6). The mean SCL-90 of the BC-group was significantly higher (p<0.05) than in the control groups. All SCL subscales were significantly different (p<0.05) between the BCs and the two control groups.

There were also significant differences (p<0.05) between the three groups using the EQ-6D; the mean EQ QOL scores were respectively in the BC group 77.9 (SD 17.2), CG1 86.7 (SD 12.8), and CG 2 86.0 (SD 11.7). Also on SF-36, there were significant differences (p<0.05) between the three groups in all subscales.

**Fig 2:**
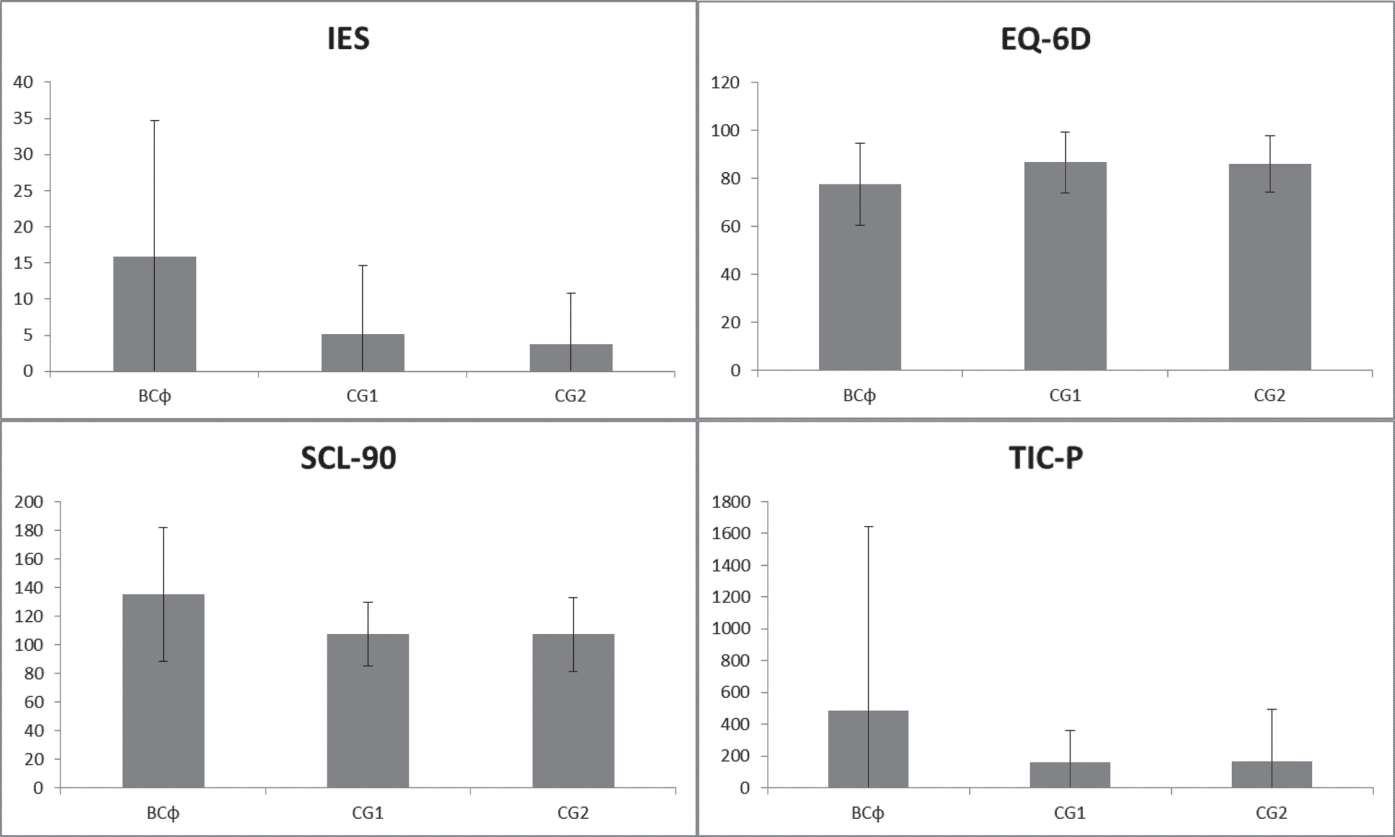
Schematic overview of mean scores of the IES, SCL-90, EQ-6D and TIC-P per subgroup.

Significant differences (p<0.05) were also found in direct medical costs consumed over the last six months between the three groups using the modified TIC-P. Mean costs of direct medical care were respectively in the BC group € 487 (SD 1154), CG1 € 162 (SD 197), and CG2 € 166 (SD 329). The mean scores (all significantly different) of the IES, SCL-90, EQ-6D and TIC-P are presented in Fig. 2.

The results of the Pearson correlation are described in Table 4, in which we analyzed the overall group and the 3 subgroups. The quality of life (EQ-6D) was negatively associated with the total scores of SCL-90 (r -.62; p<0.05) and IES (r -.48; p<0.05). A lower score on QOL was related to higher levels of distress and traumatic stress. Associations for the negative subscales of PDRS pointed out that higher QOL scores were associated with less negative attitudes on the scales WN, FN, and PN (WN = r -.18 p<0.05; FN = r -.39 p<0.05; PN = r -.23 p<0.05).

**Table 4:** Overall and subgroup relations between quality of life, distress, traumatic stress, and post deployment reintegration.

Variable	Overall	BC	CG1	CG2
Correlation (r)	N = 188	N = 62	N = 53	N = 73
IES*	-.48ɸ	-.56ɸ	-.38ɸ	-.13
INT	-.41ɸ	-.45ɸ	-.39ɸ	-.10
AVO	-.46ɸ	-.55ɸ	-.36ɸ	-.12
HAR	-.48ɸ	-.57ɸ	-.30ɸ	-.15
31.	32.	33.	34.	35.
PDRS**	36.	37.	38.	39.
WP	.19ɸ	.50ɸ	.02	.19
WN	-.18ɸ	-.11	-.07	-.20
FP	.06	.22	-.06	.01
FN	-.39ɸ	-.38ɸ	-.25	-.40ɸ
PP	-.02	.19	-.24	.03
PN	-.23ɸ	-.16	-.13	-.22
40.	41.	42.	43.	44.
SCL-90	-.62ɸ	-.62ɸ	-.60ɸ	-.53ɸ
45.	46.	47.	48.	49.
SF-36***	50.	51.	52.	53.
PF	.41ɸ	.28ɸ	.44ɸ	.41ɸ
SF	.59ɸ	.51ɸ	.51ɸ	.66ɸ
RP	.47ɸ	.43ɸ	.48ɸ	.37ɸ
RE	.55ɸ	.63ɸ	.53ɸ	.28ɸ
MH	.55ɸ	.54ɸ	.51ɸ	.48ɸ
VT	.60ɸ	.55ɸ	.64ɸ	.53ɸ
BP	.51ɸ	.56ɸ	.30ɸ	.39ɸ
GH	.60ɸ	.52ɸ	.66ɸ	.59ɸ

BC indicates battle casualty

CG1: control group 1

CG2: control group 2

n: number.significant difference (p <0.05) using the Pearson’s r correlation test.

*Subscales IES-R

INT: intrusion

AVO: avoidance

HAR: hyper arousal.

**Subscales PDRS

WP: Work positive

WN: Work negative

FP: Family positive

FN: Family negative

PP: Personal positive

PN: Personal negative.

***Subscales SF-36

PF: Physical functioning

SF: Social functioning

RP: Role physical

RE: Role emotional

MH: Mental health

VT: Vitality

BP: Bodily pain

GH: General Health.

## Discussion

This study represents the first systematic cross-sectional survey using structured questionnaires to evaluate the long-term follow-up of (Dutch) battle casualties. Ninety percent of BCs reported to be still in active duty. The QOL of BCs showed a clear reduction at the five-year follow-up, and traumatic stress levels differed significantly across the BC group compared with the control groups. The distress levels were significantly higher, and care consumption was three times higher in the BC group compared to the control groups. Correlational analysis showed that QOL was negatively associated with the total scores of SCL-90 and IES. A lower score on QOL was related to a worse outcome on distress, traumatic stress, and post deployment reintegration. The association of traumatic stress and distress levels with QOL provides an opportunity and advocates for continued interventions to manage these elevated stress levels, in order to further improve the QOL. Interestingly, long-term outcomes in the BC group were not associated with mechanism or type of injury. There was a significant relation in the BC group regarding rank and QOL. This relation was not significant for AD, ISS, and age (borderline non-significance). In our study, the lower ranked reported higher levels of distress, which may be related to higher exposure to combat stress. Focus on stress coping behavior in these groups may improve their QOL. Scott and colleagues [22] concluded that the most influential factors contributing to a patient’s QOL depended on a patient’s demographic status, socioeconomic background, and mental health. This is in line with our earlier studies in which we identified the possible protective effects of team bonding and social support network in stress coping behavior [2]. The association of lower QOL and SCL-90/IES scores could be explained by on-going effects of the initial impact (e.g. uncertainty about future, ongoing rehabilitation, and surgical treatment) after sustaining combat injury. The costs of direct medical care over the last six months, five years after sustaining combat injuries, were three times higher in the BCs compared to the control groups. Also, care consumption in the subgroup of lower extremity injuries (single and combined) was significantly higher. Including the costs of the first four years would, very likely, drastically increase the costs in the BC group. It sounds quite intuitive that combat injuries are a predictor for a lower QOL, and that assessment of the repatriated BCs, without the RTD BCs, might suggest that certain types of injury (e.g. extremity injuries) would score worse. However, when we focus on the repatriated BCs this could likely lead to eye-catching results, but with the introduction of bias [8]. In future research it could be helpful to construct one ‘sum score’ for the five used questionnaires to assess the correlation of QOL with demographics (age, rank, ISS, and AD). The majority of service members transition from an armed conflict to regular life in a seamless manner, but some struggle to find their place when leaving a highly violent theatre [23]. This study tried to outline areas of anticipated difficulty in the reintegration process, in order to alert (mental) health care providers to specific areas that could be problematic in treatment of BCs. Building on the influential factors of other studies [22–23], early identification in combination with active unit involvement and proper family rehabilitation may sustain the QOL of a BC. Other research has shown that the most important supporting factor after sustaining a battle injury seems to be peer mentoring and easy access to professional help for the injured service members and their direct social support network. Identifying predisposing factors, such as severity and type of injury, in combination with effective, low impact screening tools [23–25], could be effective as early warning system for extra mental support. In addition to early interventions in the first months after sustaining their battle injuries, we should also aim for long-term availability of support mechanisms. Our NATO coalition partners describe the same challenges [26–28]; cross-pollination seems a good opportunity to collectively improve our aftercare programs.

One of the limitations of this study is its observational character, and although associations between study variables and outcomes were determined, these associations should not be misconstrued as a cause-and-effect relationship. Also, there are some potential confounding variables that must be acknowledged. Secondly, this is a single time point retrospective study, where ideally we would have performed multiple time point assessments to assess trajectory development of the present outcomes through time. Thirdly, the questionnaires and cut off values used are only tools, and might have resulted in under- or overestimation of the (post) deployment impact. Fourthly, the relatively low numbers could be reason for limited significant outcome in regression analysis in the BC group. Semi structured interviews could be a good instrument to exploit the BC group in depth. Self-perceived aftercare requirements per subgroup of BCs might be helpful in developing a tailor made recovery program. Interestingly, the relatively low response rate (~20%) in the RTD BCs, and the responses and interactions during our visits to the Dutch wounded service members institutions [29–30], could indicate that many RTD BCs do not identify themselves as a BC. This is potentially fueled by the fact that most of them were not decorated with the wounded soldier cross.

Although reports from previous armed conflicts have been published, this study is the first to evaluate long-term patient-based outcomes in BCs in comparison to an equal group of non-injured service members from same the combat zone.

In conclusion, the QOL, the long-term impact of events, and the current distress levels were significantly worse when comparing the BC group with the control groups. Consumption of care was, five years after the injury, still three times higher among the BCs. The results of this analysis are meant to provide novel insight into management and long-term outcomes of BCs. The association of traumatic stress and distress levels with QOL, provides a window of opportunity and advocates for sustained interventions to manage these raised stress levels, in order to further improve the QOL. Future analyses in NATO perspective could help to identify modifiable factors that, hopefully, will improve outcomes among all BCs.
